# Supplementary material for: 1-Hydroxy-2(1H)-pyridinone-Based Chelators with Potential Catechol O-Methyl Transferase Inhibition and Neurorescue Dual Action against Parkinson’s Disease
Source: Molecules. 2022 Apr 28;27(9):2816. doi: 10.3390/molecules27092816 (PMC9101691; doi:10.3390/molecules27092816)
Supplement: Supplementary file 1 [file molecules-27-02816-s001.zip › molecules-1600863-supplementary.pdf]

Supplementary Materials for

1-Hydroxy-2(1H)-pyridinone-Based Chelators with Potential Catechol O-Methyl Transferase Inhibition and Neurorescue Dual Action against Parkinson's Disease

Joseph C. J. Bergin [1], Kean Kan Tan [1], Anya K. Nelson [1], Cristina-Andreea Amarandei [2], Véronique Hubscher-Bruder [2], Jérémy Brandel [2], Varvara Voinarovska [3], Annick Dejaegere [3], Roland H. Stote [3] and David Tétard [1,*]

[1] Department of Applied Sciences, Faculty of Health and Life Sciences, Northumbria University, Newcastle upon Tyne NE1 8ST, UK; joecjbergin@gmail.com (J.C.J.B.); tankeankiat@hotmail.com (K.K.T.); anyanelson98@outlook.com (A.K.N.)

[2] Université de Strasbourg, CNRS, IPHC UMR 7178, F-67000 Strasbourg, France; amarandei@unistra.fr (C.-A.A.); veronique.hubscher@unistra.fr (V.H.-B.); jbrandel@unistra.fr (J.B.)

[3] Institut de Génétique et de Biologie Moléculaire et Cellulaire (IGBMC), Institut National de La Santé et de La Recherche Médicale (INSERM), U1258/Centre National de Recherche Scientifique (CNRS), UMR7104/Université de Strasbourg, 67404 Illkirch, France; varvara.voinarovska@helmholtz-muenchen.de (V.V.); annick@igbmc.fr (A.D.); rstote@igbmc.fr (R.H.S.)

* Correspondence: david.tetard@northumbria.ac.uk

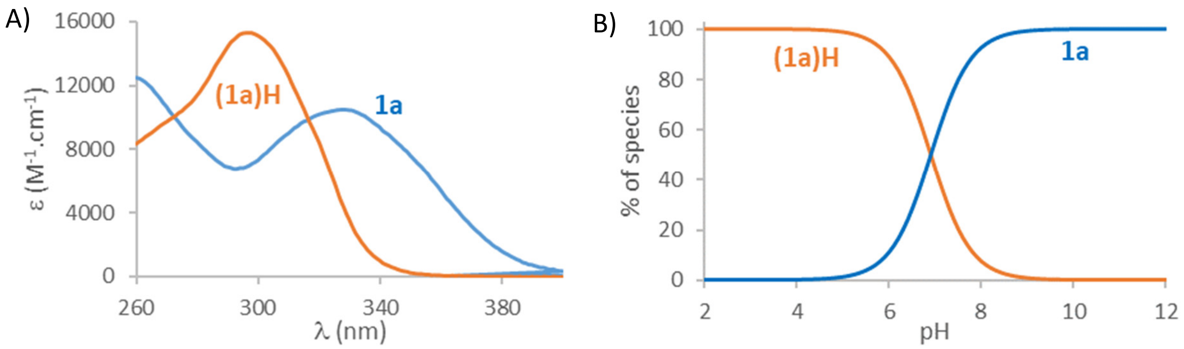

Figure S1. (**A**) Electronic spectra of the protonated species of ligand **1a** and (**B**) distribution curves of the protonated species of ligand **1a** ([**1a**] = 1.00 × 10^{-4} M). Solvent: MeOH/H_2O (80/20 w/w), I = 0.1 M ($NaClO_4$), T = 25.0 °C.

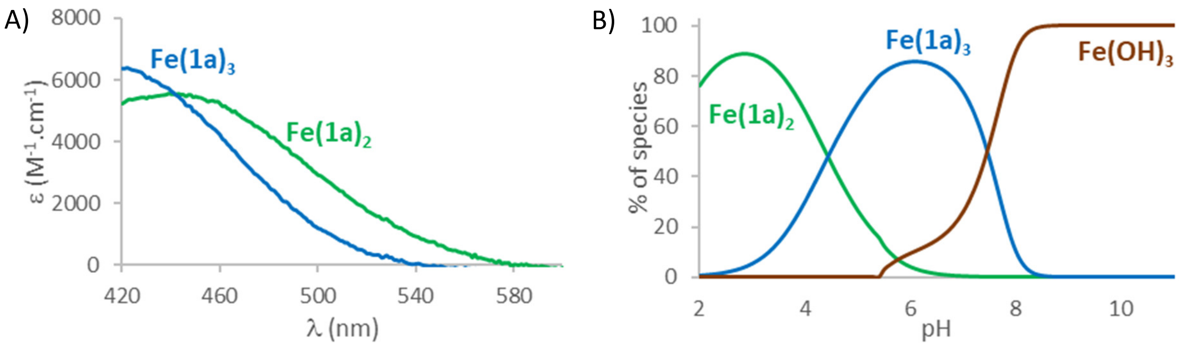

Figure S2. (**A**) Electronic spectra of Fe^{3+} complexes of **1a** and (**B**) distribution curves of Fe^{3+} complexes of **1a** relative to Fe^{3+} ([**1a**] = 1.00 × 10^{-4} M, [Fe^{3+}] = 3.20 × 10^{-5} M). Solvent: MeOH/H_2O (80/20 w/w), I = 0.1 M ($NaClO_4$), T= 25.0 °C.

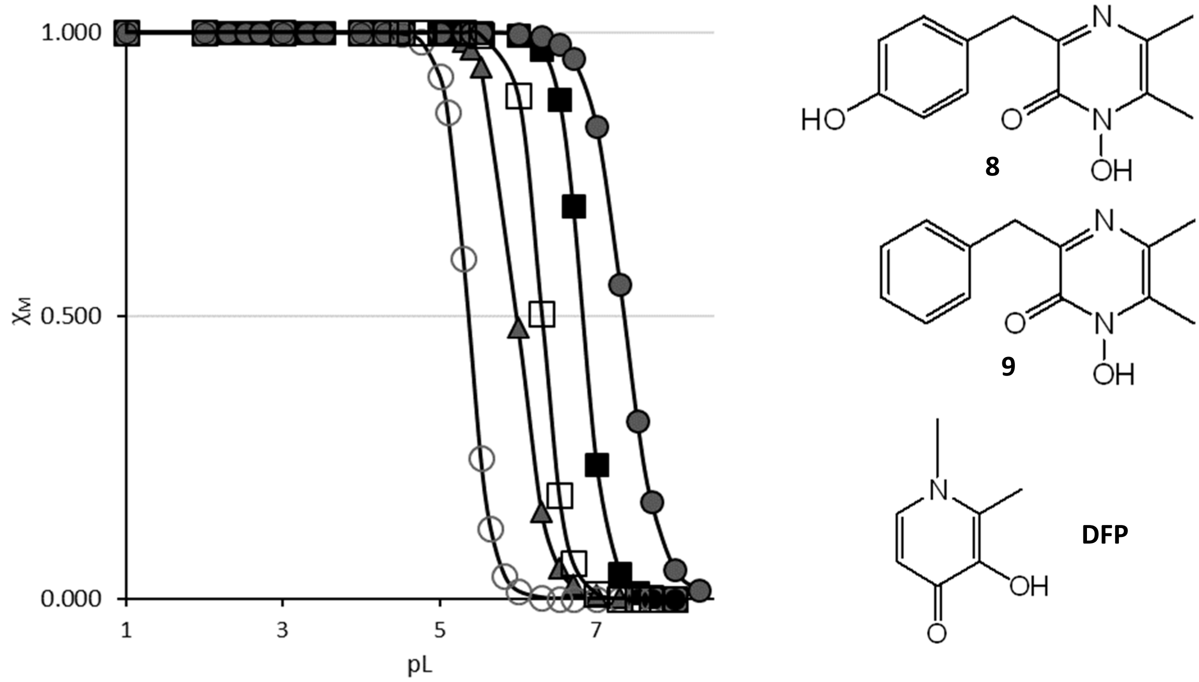

Figure S3. Sequestration diagrams towards Fe^{3+} at pH = 7.4 of ligands (from left to right), **1a** (O), **6c** (▲), **1b** (□), DFP (■), **6d** (●) and [Fe^{3+}] = 10^{-9} M, T = 25.0 °C, I = 0.1 M ($NaClO_4$).

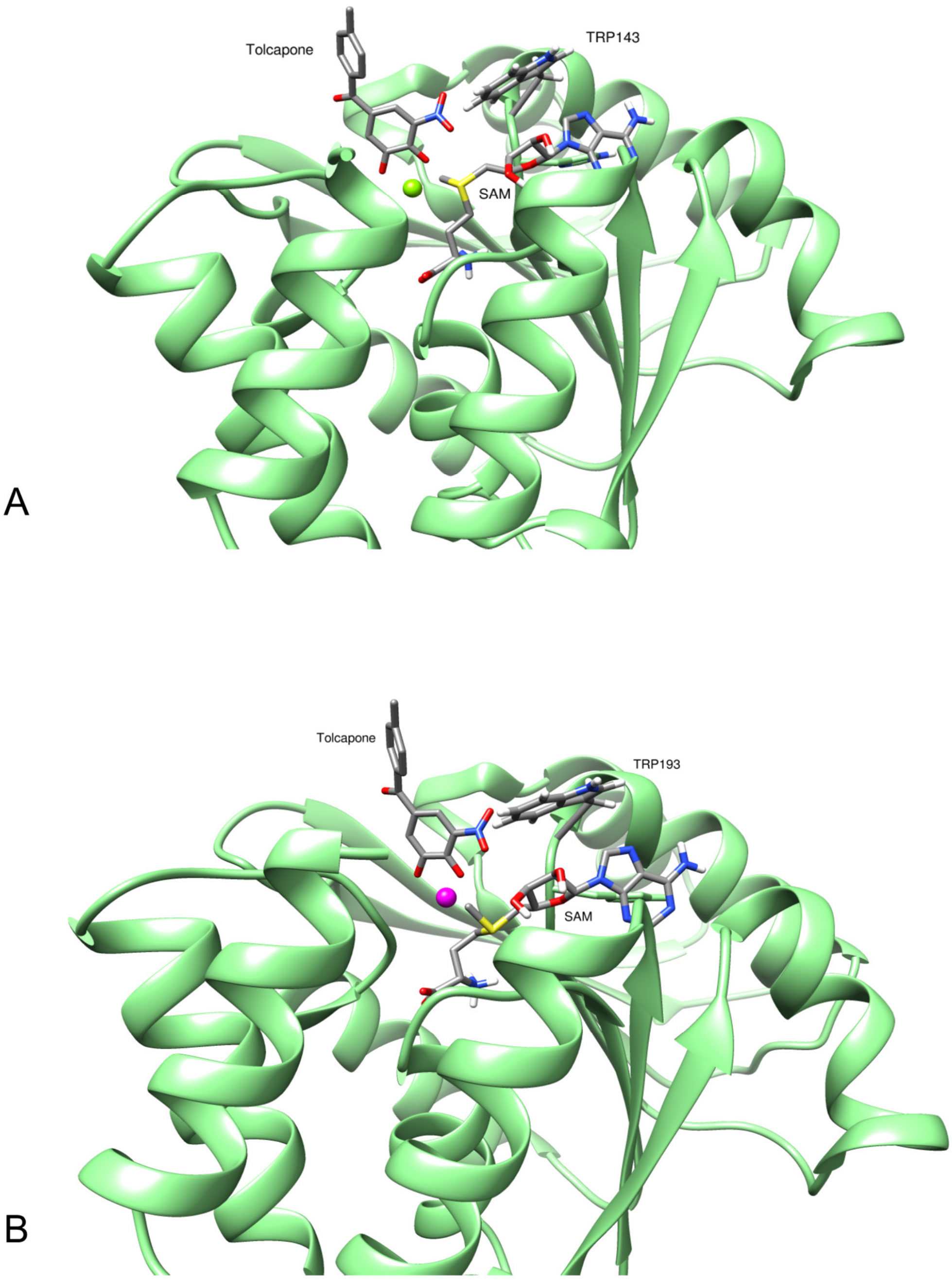

Figure S4. Starting conformations for the simulations of rat and human COMT in complex with Tolcapone, (**A**) rat COMT (PDBID 3S68), (**B**) human COMT (based on PDBID 5lsa)

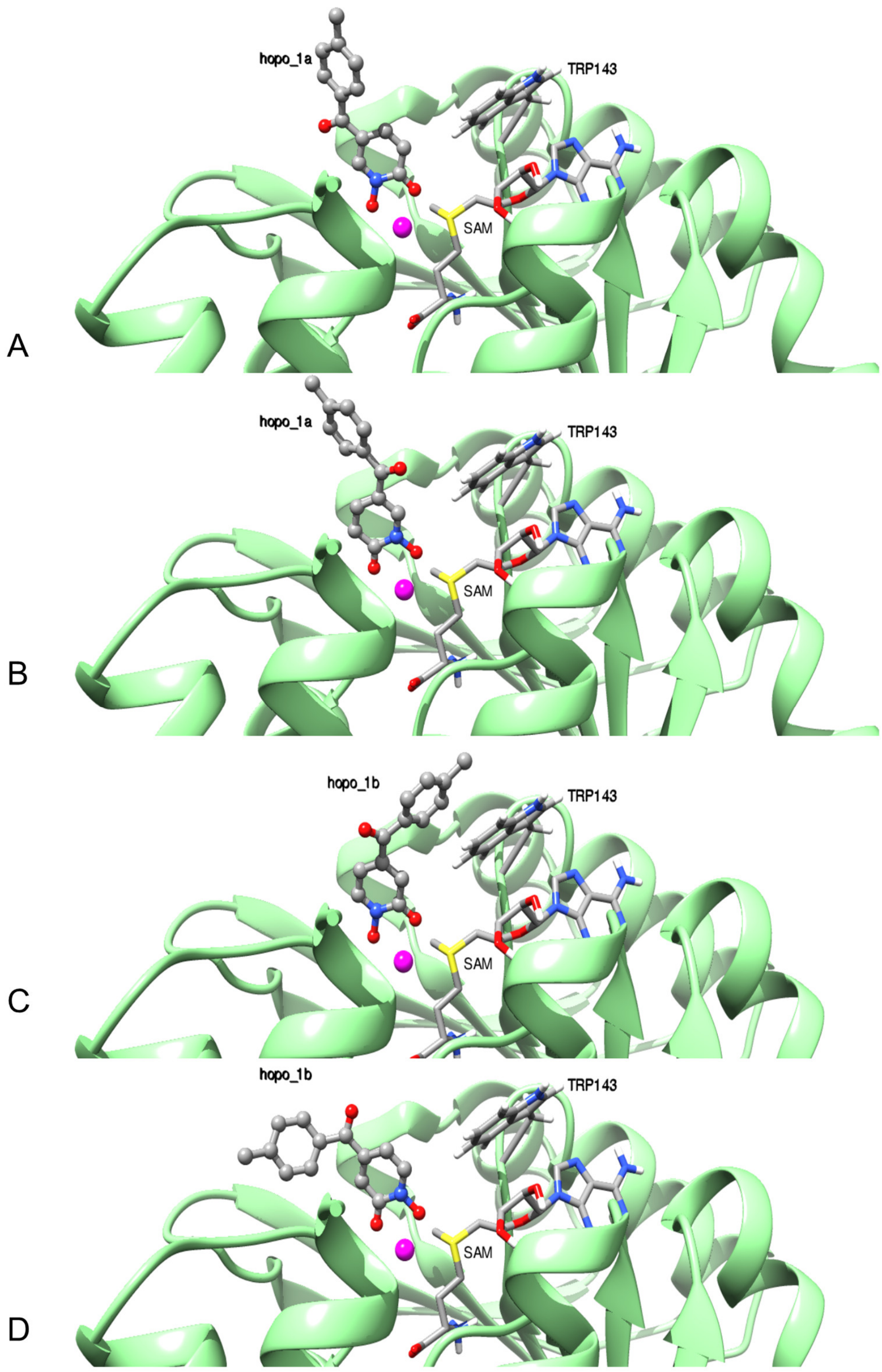

Figure S5. Starting conformations for the simulations of the rat COMT/HOPO complexes, based on PDBID 3S68. (**A**) **1a**, (**B**) **1a** with NO pointing toward SAM, (**C**) **1b**, D) **1b** with NO pointing towards SAM

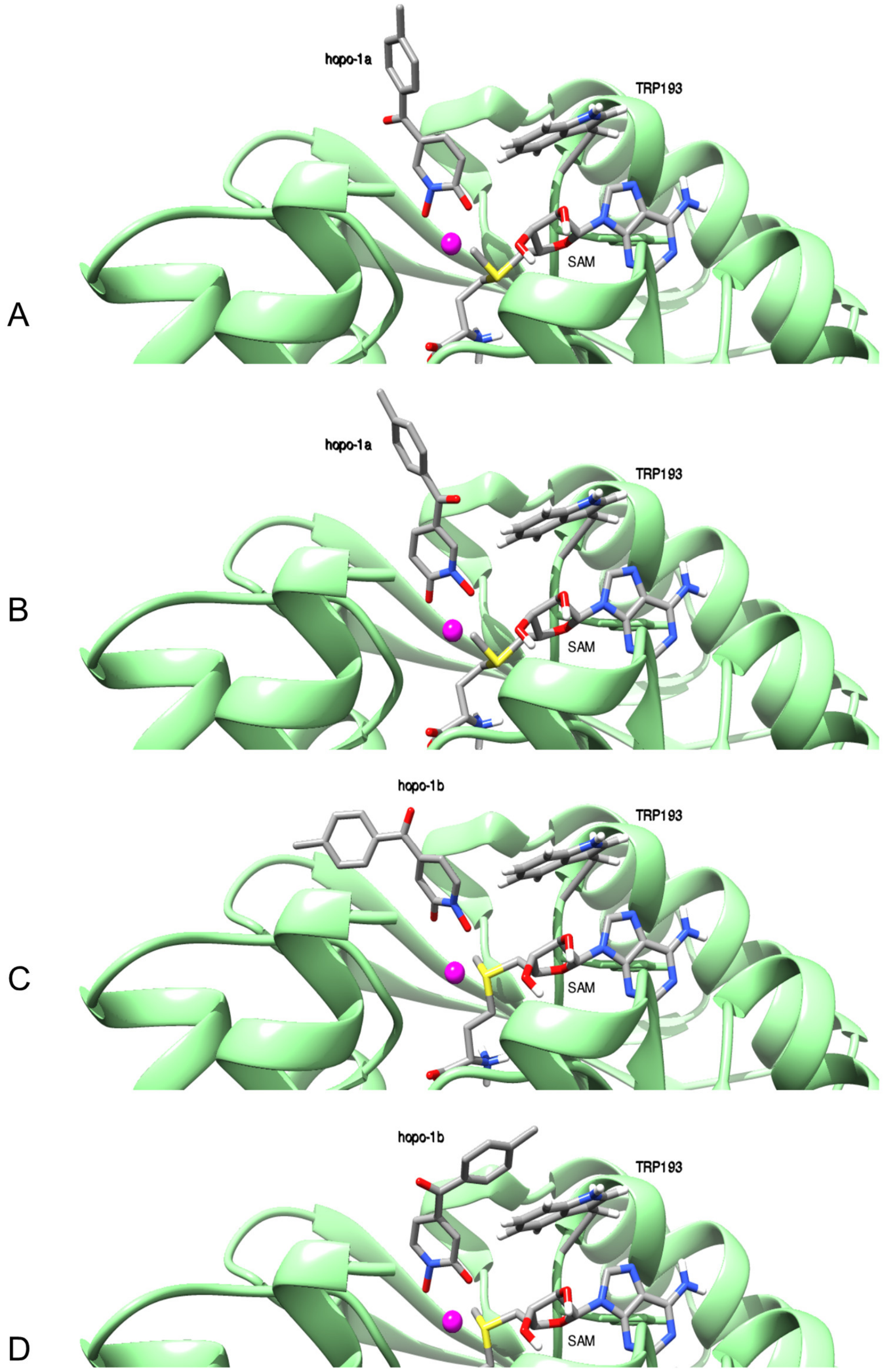

Figure S6. Starting conformations for the simulations of the human COMT/HOPO complexes, based on PDBID 3S68. (**A**) **1a**, (**B**) **1a** with NO pointing toward SAM, (**C**) **1b**, D) **1b** with NO pointing towards SAM

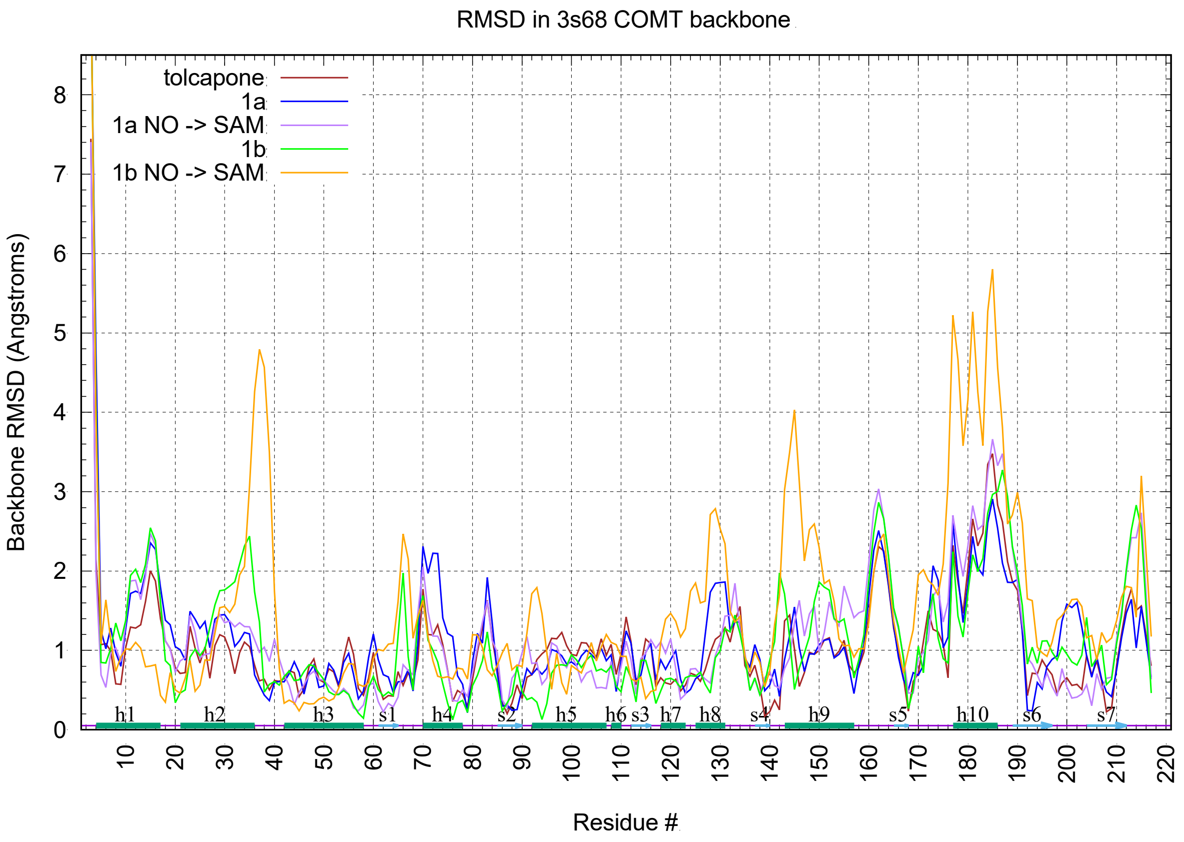

Figure S7. Backbone RMSD by-residue for average structures of the different rat COMT complexes calculated at the end of the simulation (averaged over 10ns)

A)

B)

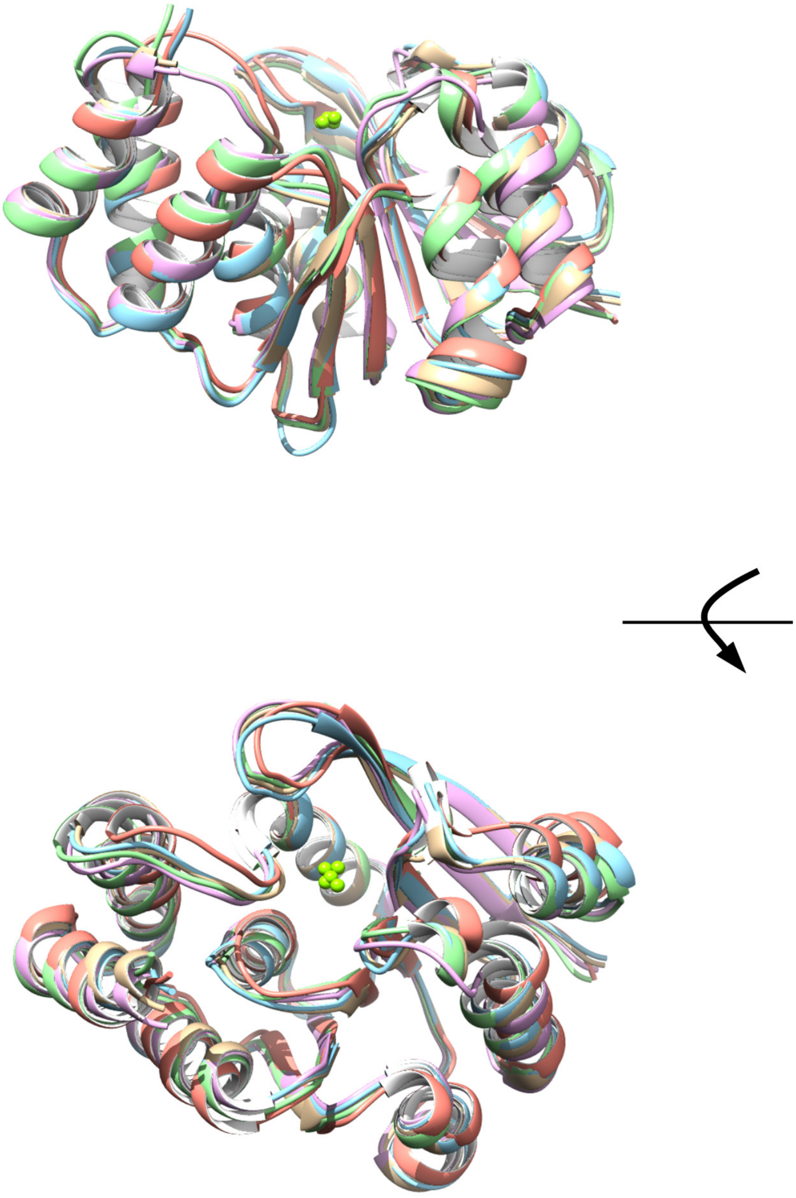

Figure S8. Superposition of average structures from the end of the MD simulations of the complexes of rat COMT, (**A**) a side view and (**B**) top-down view obtained by rotating 90° to look down at the ligand binding site with the Mg^{+2} ion shown as a green sphere; colour legend, in complex with tolcapone in brown, with hopo-1a in blue (see Fig. 6a) , with hopo-1a_NO->SAM in magenta (see Fig. 6b), with hopo-1b in green (see Fig. 6c), with hopo-1b_NO->SAM in orange (see Fig. 6d)

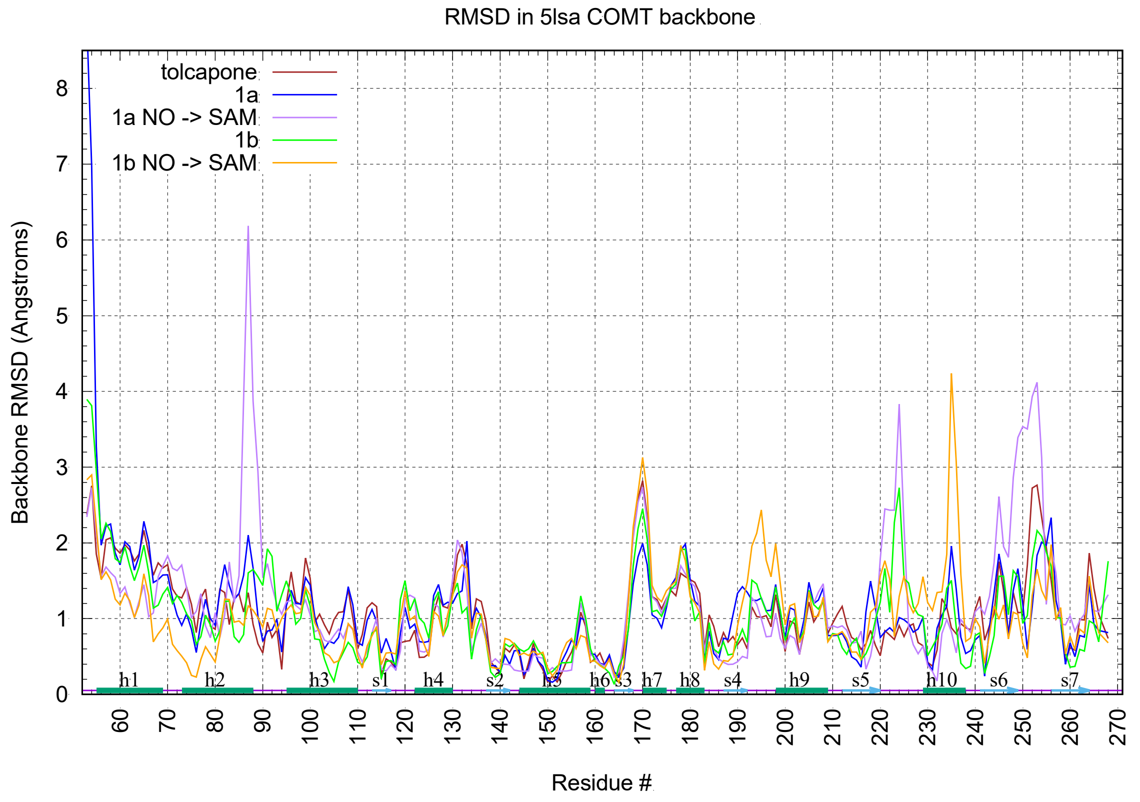

Figure S9. Backbone RMSD by-residue for average structures of the different human COMT complexes calculated at the end of the simulation (averaged over 10ns)

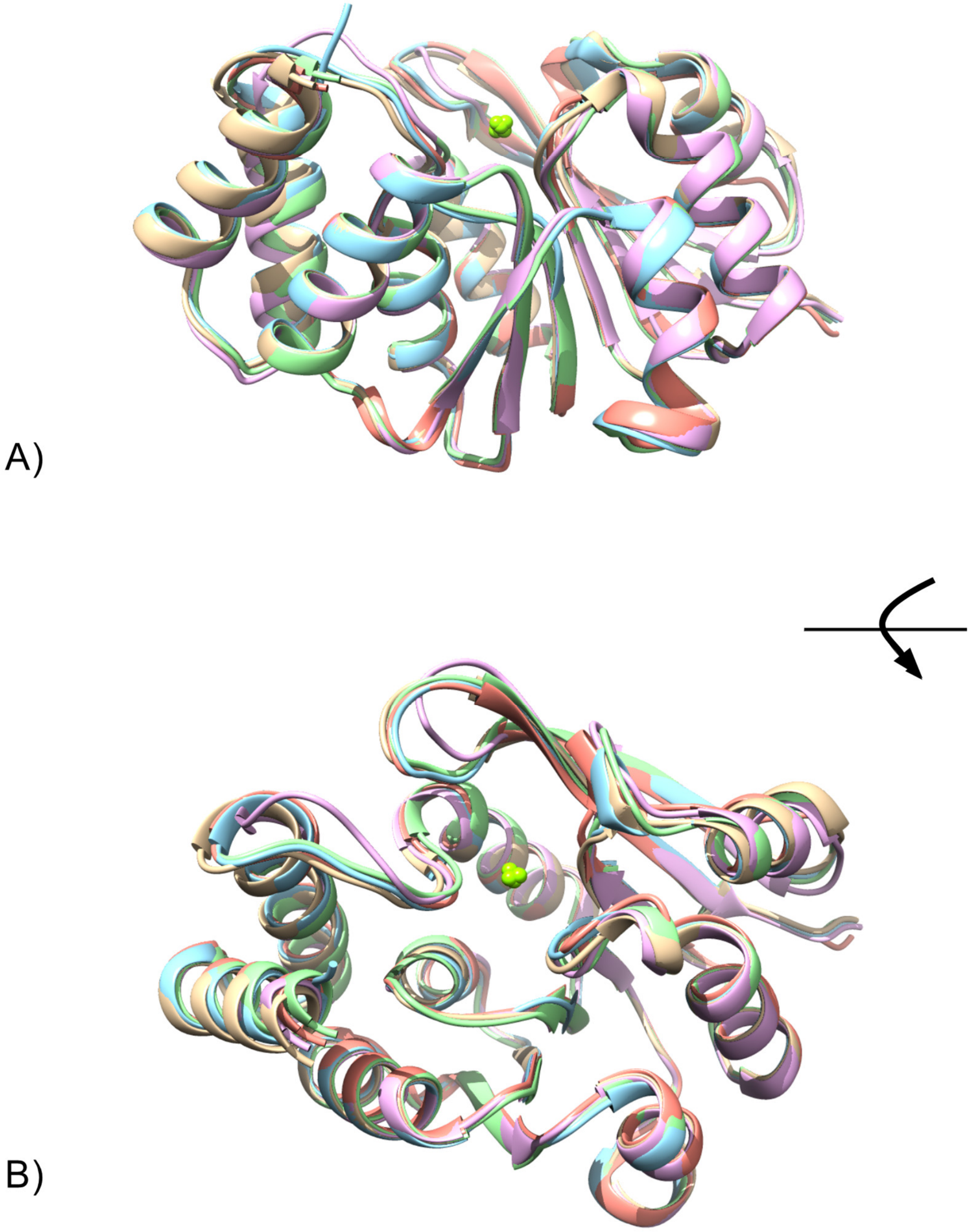

Figure S10. Superposition of average structures from the end of the MD simulations of the complexes of human COMT, (**A**) a side view and (**B**) top-down view obtained by rotating 90° to look down at the ligand binding site with the Mg^{+2} ion shown as a green sphere; colour legend, with complex with tolcapone in brown, with hopo-1a in blue (see Fig. 6a) , with hopo-1a_NO->SAM in magenta (see Fig. 6b), with hopo-1b in green (see Fig. 6c), with hopo-1b_NO->SAM in orange (see Fig. 6d)

Table S1. Binding Free energy scores with absolute scores with standard deviations and differences between doubly protonated tolcapone score and the scores of the other compounds and orientations

Species	Ligand Orientation	◎$G_{binding}$ (kcal/mol)	◎$G_{binding}$ (Tol) -◎$G_{binding}$ (Compound)
Rat	Tolcapone doubly deprotonated (Fig 5a)	-30.9 (±9.6)	0.0 (±9.6)
Rat	Tolcapone singly deprotonated (Fig. 5b)	-13.0 (±5.4)	17.9 (±5.4)
Rat	HOPO-1a =O→SAM (Fig 6a)	+4.7 (±5.9)	35.6 (±5.9)
Rat	HOPO-1a N-O-→SAM (Fig 6b)	-28.8 (±5.1)	2.1 (±5.1)
Rat	HOPO-1b N-O-→SAM (Fig 6c)	-20.4 (±5.7)	10.5 (±5.7)
Rat	HOPO-1b =O→SAM (Fig 6d)	-27.6 (±3.9)	3.3 (±3.9)
Human	Tolcapone doubly deprotonated (Fig 1a)	-23.6 (±5.4)	0.0 (±5.4)
Human	Tolcapone singly deprotonated, (Fig 1b)	-17.8 (±6.8)	5.8 (±6.8)
Human	HOPO-1a =O→SAM (Fig 6a)	+7.8 (±5.1)	31.4 (±5.1)
Human	HOPO-1a N-O-→SAM (Fig 6b)	-28.3 (±5.3)	-4.7 (±5.3)
Human	HOPO-1b N-O-→SAM (Fig 6c)	-0.5 (±4.9)	23.1 (±4.9)
Human	HOPO-1b =O→SAM (Fig 6d)	-7.1 (±4.7)	16.5 (±4.7)
